# Rare Occurrence of Methicillin-Resistant *Staphylococcus aureus* CC130 with a Novel *mec*A Homologue in Humans in Germany

**DOI:** 10.1371/journal.pone.0024360

**Published:** 2011-09-08

**Authors:** Christiane Cuny, Franziska Layer, Birgit Strommenger, Wolfgang Witte

**Affiliations:** Wernigerode Branch, Robert Koch Institute, Wernigerode, Germany; Charité-University Medicine Berlin, Germany

## Abstract

MRSA CC130 containing the *mec*A homologue *mec*A_LGA251_ were reported from the UK and from Denmark so far from cattle and humans. Here we report on 11 MRSA CC130 among a sample of 12691 isolates of human origin collected from January 2006 until June 2011. MRSA CC130 grew insufficiently on chromogernic agar plates for detection of MRSA; the agglutination test for presence of PBP2a was negative. We designed primers for specific detection of *mec*A_LGA251_ as well as for concomitant detection of both, *mec*_LGA251_ and *mec*A. As already described, the isolates exhibited *spa*-types t843, t1736, and t1773. The *ccr*A homologue indicated the presence SCC*mec*_XI_. When subjected to further characterization by means of a commercially available microarray the isolates were negative for *sak chp*, and *scn*, and as expected positive for *hla*, untruncated *hlb*, and *hld*. They furthermore contained *edin*B, *aur*, *slp*A, *slp*B, *slp*E. From genes coding for surface and cell wall associated products the *ica*-operon, *cap*8, *clf*A, *clf*F, *ebp*S, *fnb*A, *fnb*B, *sdr*C were detected but not *cna*. The isolates were negative for enterotoxin genes and *tst*, as well as for *eta*, and *etb*; *agr*-type was III.

## Introduction

Nosocomial infections with methicillin resistant *Staphylococcus aureus* (MRSA) became an infection control problem worldwide during the past 20 years. They are mainly associated with hospital associated, clonal lineages (HA-MRSA) which have a pronounced capacity for spread in and among hospitals [1]. By the end of the 1990s MRSA emerged in parallel in the community independent upon hospitals. Community associated MRSA (CA-MRSA) obviously evolved separately from HA-MRSA, the majority of them represent clonal lineages different from HA-MRSA [2]. Since 2005 the emergence of MRSA in animals, in particular in livestock, focussed attention (LA-MRSA). The predominant clonal lineage ST398 was at first found as a colonizer in conventionally raised pigs, later on in other livestock such as veal calves and poultry, and finally in humans where it is not only a colonizer but infrequently the cause of infections, too [3]. The first demonstration of MRSA from mastitis in cattle dates back to 1972 [4], altogether MRSA remained infrequent in ruminants [5], [6]. Earlier studies based on phenotypic characterization suggested that *S. aureus* from cattle and from humans are unrelated [7]. This was confirmed by molecular typing for clonal lineages ST97 and ST705 of bovine origin, which seem to be pandemic [8], [9]. Mastitis associated lineages ST151 and ST133 seem to be frequent in the United Kingdom (UK) and in Denmark [10], [11]. However the majority of isolates from these lineages remained methicillin susceptible. There are several reports on mastitis in cattle associated with LA-MRSA ST398 [12]. Also clonal lineages of MRSA known before from humans have been reported in association with bovine mastitis such as HA-MRSA ST5 from Japan [13], ST239 from Turkey [14] and CA-MRSA ST1 from Hungary [15] and from Korea [16]. Another example for a clonal lineage with no restricted host specificity is *S. aureus* ST130 which represents a smaller fraction among *S. aureus* from mastitis in cattle in UK and more recently also MRSA ST130 from humans have been described [17], [18]. Interestingly these MRSA contain the *mec*A homologue *mec*_LGA251_ which is not detected by PCR established for detection of *mec*A. When human isolates exhibiting MRSA phenotype but negative PCR for mecA were tested, *mec*A_LGA251_ was also detected among MRSA from humans in England and in Denmark. Here we report about the emergence of MRSA ST130 in infections in humans in Germany at low frequencies, PCR detection, and the uncertainty of detection by use of chromogenic media based on cefoxitin as selective agar.

## Materials and Methods

### Strain collection

MRSA isolates from a network of diagnostic laboratories all over Germany were sent to the German National Reference Centre for Staphylococci at the Robert Koch Institute, Wernigerode Branch, for typing and strain characterization.

The isolates were cultured on sheep blood agar and the species identification was confirmed by conventional methods. Additionally, all isolates were subjected to antibiotic susceptibility testing, *spa-*typing and PCR detection for the presence of *mec*A. A subset of selected isolates was further characterized.

No patient data were used or stored for the present study. Therefore, consent from the ethics committee was not required.

### Phenotypic characterisation

Antimicrobial susceptibility testing was performed using the broth microdilution assay as described by Deutsches Institut für Normung, DIN 58940. [19] Interpretation of the results was done according to the EUCAST standard [20]. The MIC test panel included penicillin G, oxacillin, gentamicin, erythromycin, clindamycin, ciprofloxacin, moxifloxacin, oxytetracycline, cotrimoxazol, rifampicin, fusidic acid, fosfomycin, linezolid, mupirocin, daptomycin, tigecyclin, vancomycin, and teicoplanin. Furthermore all isolates were checked for susceptibility against oxacillin/sulbactam by a tube test as described previously [21].

Test for growth on chromogenic selective agar plates: Colonies grown on blood agar plates after overnight incubation were suspended in 0.9% NaCl solution with a turbidity corresponding to the 0.5 McFarland turbidity standard. This suspension and appropriate dilutions were inoculated in parallel onto sheep blood agar plates (Mueller Hinton agar containing sheep blood, OXOID) and onto chromogenic agar plates from three different manufacturers (chromID™ MRSA, bioMerieux; Brilliance™ MRSA, Oxoid; chromagar™ MRSA, Becton Dickinson). Growth was recorded after overnight incubation at 37±1°C. Efficiency of plating was calculated as the proportion of colony forming units on selective agar plates in comparison to blood agar plates.

Phenotypic test for the expression of PBP2a: The Slidex Staph MRSA test kit from bioMerieux was used as recommended by the manufacturer.

### Genotypic characterisation

Primers used for PCR-based *mec*A-detection correspond to [22]. Primers were designed for the detection of *mec*_LGA251_ by choosing regions of low homology in the genome of MRSA LGA251 (accession-no. FR821779, EMBL database). Primers for the concomitant detection of *mec*A and *mec*_LGA251_ ( =  *mec*_u_) were also deduced from the sequence of the genome of MRSA LGA251 by choosing regions nearly identical to *mec*A. A PCR for the *ccr*A homologue described for SCC*mec*_XI_ in MRSA ST130 was developed by using primers deduced from the genome sequence of MRSA M10/0061 (accession-no. FR823292, EMBL database) (Table 1). The sequence of the respective PCR product shared >99% identity with the corresponding sequence in FR823292 and homologies <91% with *ccr*A of other SCC*mec* elements reported so far. The cycling scheme for the PCR reactions were 95°C for 2 min followed by 30 cycles with 94°C for 30 sec; 30 sec with the respective annealing temperature, 72°C for 30 sec; and finally 72°C for 4 min.

**Table 1:** Primers used for amplification and sequencing of *mec*A, *mec*_LGA251_, *mec*_u_ and *ccr*A_XI_.

Primer	Sequence (5′-3′)	PCR product (bp)	T_ann_	Target localisation	Reference
*mec*A f	TGGCTCAGGTACTGCTATCCAC	776	60°C	1038–1059 in Y00688	[22]
*mec*A r	AGTTCTGCAGTACCGGATTTGC			1814–1793 in Y00688	
*mec*_LGA251_ f	GCTCCTAATGCTAATGCA	304	50°C	36321–36338 in FR821779	This study
*mec*_LGA251_ r	TAAGCAATAATGACTACC			36625–36608 in FR821779	
*mec*_u_ f2	ATTTGTCTKCCAGTTTC	728	48°C	35907–35823 in FR821779	This study
*mec*_u_ r2.1	TCACCAGGTTCAACRCA			36551–36534 in FR821779	
*ccr*A_XI_ f	TTTGGATTGTATGGTTRGA	261	50°C	12271–12288 in FR823292	This study
*ccr*A_XI_ r	CTCAAACGGACATCATCA,			12532–15215 in FR823292	

For *spa*-typing the polymorphic X-region of the protein A gene (*spa*) was amplified and sequenced according to the Ridom StaphType standard protocol (www.ridom.com). The resulting *spa*-types were assigned by using the Ridom StaphType software package (Ridom GmbH, Würzburg, Germany). The BURP algorithm, implemented in the most recent Ridom StaphType software version, was used for cluster analysis of *spa*-types [23]. Primers used for MLST correspond to the protocol as described previously [24], with the exception of the forward primer for *tpi*, where the following primer was used: tpif 5′-GCATTAGCAGATTTAGGCGT-3′.

SCC*mec* elements of types I to IV were characterized by using a PCR approach, including a combination of different PCRs as described (www.staphylococcus.net).

Selected isolates (08-02742, 09-01300, 09-02494, 10-00991, 10-01051, 10-01981, 10-02462-1) were further genotyped with the Identibac S. aureus Genotyping Test System (Alere Technologies GmbH) and the procedures recommended by the producer. This DNA microarray was initially developed and described by Monecke et al. [25]. It covers 334 target sequences (221 distinct genes represented by non polymorphic probes and a number of allelic variants) including taxonomic markers, SCC*mec*, capsule and *agr* group typing markers, resistance genes, exotoxins, and MSCRAMM genes. The hybridisation pattern was analyzed with the respective instrument and software (ArrayMate, Alere Technologies GmbH).

## Results

### Detection of MRSA ST130

The isolates reported here attracted attention by exhibiting resistance to oxacillin (MIC 8 mg/l) and to oxacillin/sulbactam (tube test) but negative PCR for *mec*A. MIC's for cefoxitin were 4 mg/l for 9 of the isolates and 8 mg/l for 2 of them. The 11 isolates MRSA ST130 investigated were only weakly able to grow on commercially available selective agar plates for MRSA. At least ChromID™ MRSA agar permitted growth of 8 of these isolates (Table 2).

**Table 2:** Efficiency of plating of MRSA ST130 on chromogenic agar plates in comparison to blood agar plates (% as proportion of colony forming units on blood agar plates).

Proportion of colony forming units growing on chromogenic agar plates selective for MRSA in comparison to blood agar plates					
Number ofStrains	ChromID™(bioMerieux)	Number of strains	Brilliance™ MRSA(Oxoid)	Number ofstrains	Chromagar™ MRSA(Becton-Dickinson)
8	50–100%	4	50–100%	1	80%
3	0,1–0,2%	1	10%	4	5–10%
		6	0,01%	6	0,01–0,05%

All of the 11 isolates gave a negative result when subjected to phenotypical detection of PBP2a by means of the Slidex MRSA detection kit (bioMerieux). PCR amplification of the *mec*A homologue *mec*_LGA251_ by use of primers deduced from the sequence of MRSA ST130 strain LGA251 resulted in an amplimer of the corresponding size (304 bp) and nucleotide sequence. PCR for the *ccr*A homologue typical for SCC*mec*_XI_ confirmed the presence of this SCC*mec* element.

PCR for covering both, *mec*A and *mec*_LGA251_: For establishing this PCR a sequence of high homology has been chosen for primer design. This PCR gave positive results for the major lineages of HA-MRSA, CA-MRSA, LA-MRSA ST398, and MRSA isolates ST130 (Table 3).

**Table 3:** Results for PCR detection of *mec*A in major MRSA clonal lineages and mecLGA251 in MRSA ST130 by different sets of primers.

clonal lineage, SCC*mec*, *spa*-type	number of isolates	PCR results		
		*mec*A	*mec*_LGA251_	*mec*_u_
ST5, II, IV	4	+	-	+
ST8, IV, t008	5	+	-	+
ST22, IV, t022, t032	4	+	-	+
ST36, t018	1	+	-	+
ST45, t004, t015	5	+	-	+
ST225, II, t003	3	+	-	+
ST228, II, t001	4	+	-	+
ST239, III, t037	3	+	-	+
ST247, I, t051	3	+	-	+
ST1, IV, t127, PVL+	1	+	-	+
ST5, V, t002, PVL+	1	+	-	+
ST8, IV, t008, PVL+	2	+	-	+
ST22, IV, t310, PVL+	2	+	-	+
ST59, IV, t216, PVL+	1	+	-	+
ST80, IV, t044, PVL+	3	+	-	+
ST152, V, t355	1	+	-	+
ST398, V, t011, t034	4	+	-	+
ST130, XI, t843, t1736, t1773	11	-	+	+

### Results from typing

The isolates exhibited *spa*-types t843, t1736 and t1773 and multilocus sequence type ST130. Besides resistance to oxacillin and to oxacillin/sulbactam only two of the 11 isolates exhibited resistance to ciprofloxacin.

### Origin of MRSA ST130

The first isolate of this MRSA lineages from infections in humans we became aware of originated from a patient suffering from a wound infection which needed surgical treatment in a hospital in Saxony Anhalt in 2006. Until 2011 9 other cases followed. Altogether MRSA ST130 were rare so far; 11 isolates among 12691 MRSA isolates from infections in humans (2006: 1 among 1514; 2007: 1 among 1972; 2008: 1 among 2287; 2009: 2 among 3168; 2010: 4 among 2853; 2011: 2 among 897).

The origin of these isolates and their characteristics are shown in Table 4.

**Table 4:** MRSA ST130: origin and characteristics.

No.	Isolate area	origin	*spa*-type	resistance phenotype
06-01833	H. Saxony Anhalt	wound infection	t1736	PEN,OXA,OXA/SUL,CIP
07-03307	H. Bavaria	wound infection	t843	PEN,OXA,OXA/SUL
08-02742	B.W.	nasal swab veterinarian	t1736	PEN,OXA,OXA/SUL
09-01300	NDH,Thuringia	dermatitis	t1773	PEN,OXA,OXA/SUL
09-02494	WP,NRW	wound infection	t843	PEN,OXA,OXA/SUL
10-00991	Rd, Schl.-H.	wound infection	t1736	PEN,OXA,OXA/SUL
10-01051	Lp. Saxony	wound infection	t1736	PEN,OXA,OXA/SUL
10-01981	Bd-L., Thuringia	wound infection after hip replacement	t843	PEN,OXA,OXA/SUL
10-02462-1	H. Saxony Anhalt	wound infection	t1736	PEN,OXA,OXA/SUL
11-00529	RZ, W.-W.	wound infection	t843	PEN,OXA,OXA/SUL,CIP,MFL
11-01497	Saxonia	nosocomial pneumonia	t1736	PEN,OXA,OXA/SUL

CIP  =  ciprofloxacin, PEN  =  benzylpenicillin, OXA  =  oxacillin, OXA/SUL  =  oxacillin/sulbactam,

MFL  =  moxifloxacin.

### Further characterization by means of microarray analysis for virulence and antibiotic resistance genes

Detailed results of the DNA microarray hybridisation are provided in Figure S1.

For genes of particular interest we obtained the following patterns:

hemolysins: *hla*+, *hlb*+, *hld*+

immune evasion: *sak-*, *chp*-, *scn*-, *aur*+, *splA*+, *splB*+, *splE*+,

matrix protein binding: *bbp*-, *can*-, *clfA*+, *clfB*+, *ebh*+, *fnbA*+, fnbB*+,*

*sdrC*+ (non polymorphic probes), *coa*+, *vwb+* (non polymorphic probe)

superantigens: *tst*-, *sea* to *seq* -

exfoliative toxins: *eta*-, *etb*-, *etd*-, *edinA*-, *edinB*+

capsule, biofilm: *cap*8+, *ica*-operon+

agr type: III

Abbreviations: *aur*: “aureolysin”, proteinase; *bbp*: bone sialoprotein binding protein; *cap*: capsule formation; *chp*: chemotaxo inhibitory protein; *clf*A, *clf*B: clumping factor (fibrinogen binding); *can*: collagen binding protein; *coa*: coagulase; *ebh*: fibronectin binding protein; *edin*: epidermal cell differentiation inhibitors; *eta*, *etb*, *etd*: exfoliative toxins; *fnb*: fibronectin binding proteins, *ica*-operon: protein involved in biofilm formation; *sak*: staphylokinase; *sea* to *seq*: staphylococcal enterotoxins; *scn*: staphylococcal complement inhibitor; *spl*: serin protease like proteins; *vwb*: non Willebrandt factor binding protein.

## Discussion

As typical for *S. aureus* from ruminants and other animal species MRSA ST130 lack immune evasion genes *sak*, *chp*, and *scn* [26]. They are contained by a phage of the phi3 family which integrates into *hlb* [27]. Corresponding to the lack of these genes MRSA ST130 isolates contain an untruncated *hlb* gene. Redundancy in *S. aureus* virulence associated proteins is common, and the function other proteins such as the aureolysin protease (*aur*) and serin protease like enzyme(s) may be sufficient. MRSA ST130 contain a number of genes coding for matrix protein binding proteins also known from *S. aureus* clonal lineages typical for humans such as *fnb*A, *fnb*B, *clf*A, *clf*B, *sdr*C, and *ebh*. A surprising similarity of immune evasion and surface protein genes of *S. aureus* from different animal hosts was reported just recently [28]. Different alleles of these genes are lineage specific and not associated with the major animal host. None of the known superantigen determinants was found in MRSA ST130, but a number of genes for staphylococcal superantigen like proteins was detected (*set*6, *set*8, *set*3, *set*5, *set*1) for which the function is not exactly known so far.

Genes coding for exfoliated toxins were absent besides *edin*B, this corresponds to the clinical picture infections with MRSA ST130 in humans recorded.

MRSA ST130 has the potential for biofilm formation (*ica*-gene cluster) and of formation of a type 8 capsule. Capsular serotype 8 is known from *S. aureus* of both, human and bovine origin [29] and capsules of this type protect against both, human and bovine neutrophils [30].

Although MRSA ST130 have not been reported from cattle in Germany so far an animal origin seems likely, this is also supported by its isolation from a nasal swab of a veterinarian in Bavaria. As reported from Denmark and from the UK MRSA ST130 is obviously able to colonize and to cause infections in both, cattle and humans (17, 18). Different from HA-MRSA and LA-MRSA the isolates were only resistant to beta lactam antibiotics, two of them in addition to fluoroquinolones. As fluoroquinolone resistance is uncommon in *S. aureus* from bovine mastitis it has probably been acquired by fluoroquinolone treatment of humans.

So far MSSA and MRSA ST130 are very rare among isolates from infections in humans. Also we did not find MSSA of this clonal lineage in healthy carriers investigated [31], [32]. Because of the low MICs for cefoxitin MRSA ST130 can be overlooked when using selective agar plates for screening at admission to hospitals. They should be considered also and verified by an appropriate PCR for the *mec*_LGA251_, when oxacillin/cefoxitin resistant but *mec*A negative isolates appear in routine clinical bacteriology.

Future studies have to show whether *S. aureus*/MRSA ST130 will to be able to disseminate among humans.

## Supporting Information

Figure S1 **Array hybridisation results for selected isolates.**
(PDF)
