# Supplementary figures and images for: Rare Occurrence of Methicillin-Resistant Staphylococcus aureus CC130 with a Novel mecA Homologue in Humans in Germany
Source: PLoS One. 2011 Sep 8;6(9):e24360. doi: 10.1371/journal.pone.0024360 (PMC3169590; doi:10.1371/journal.pone.0024360)

**Figure S1:** Array hybridisation results for selected isolates.

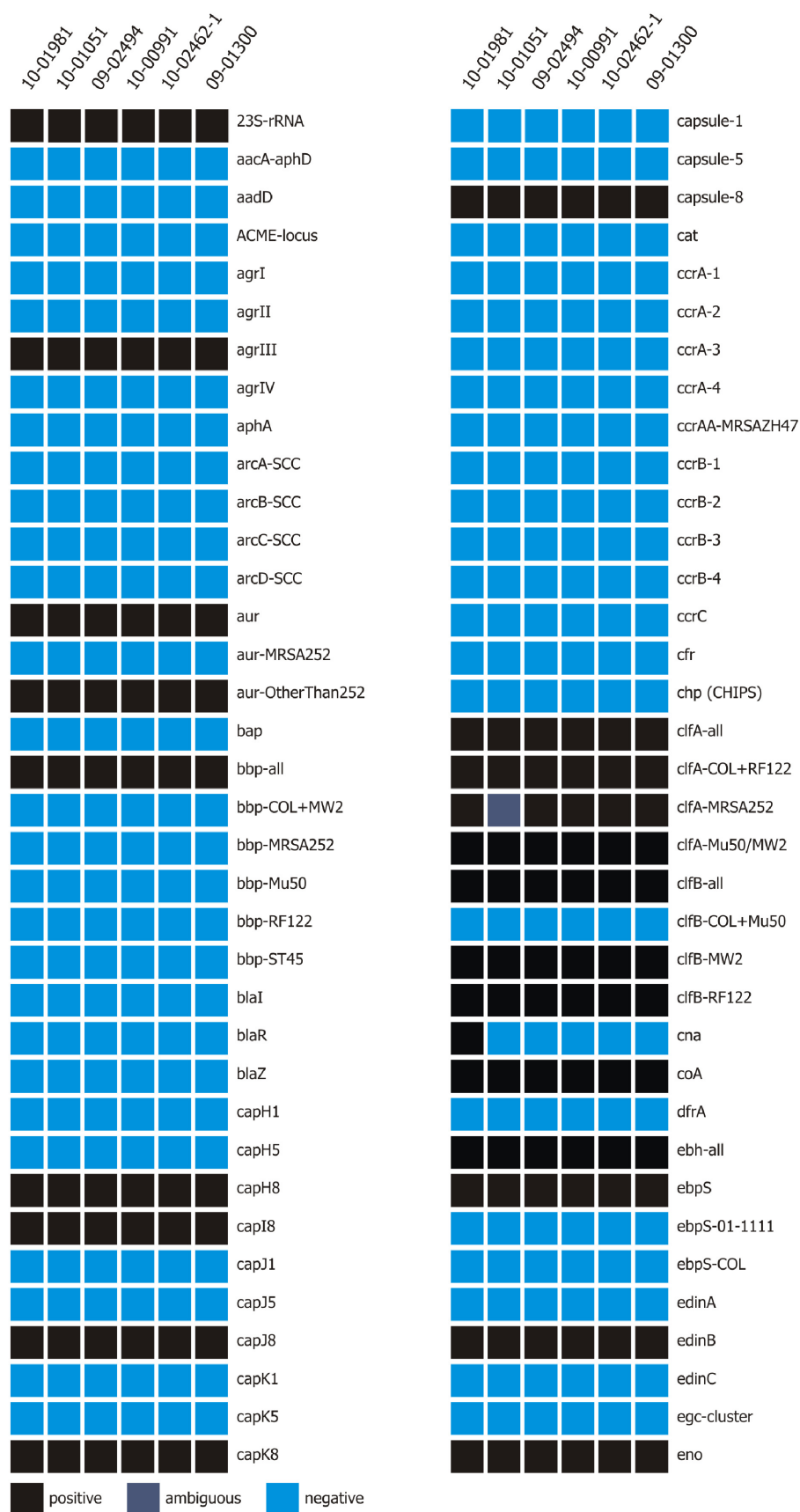

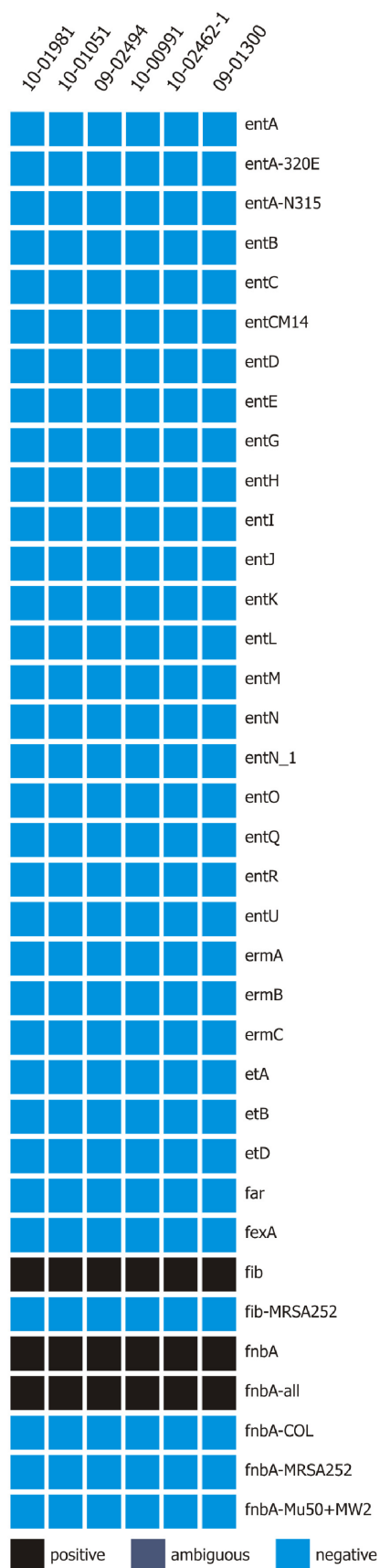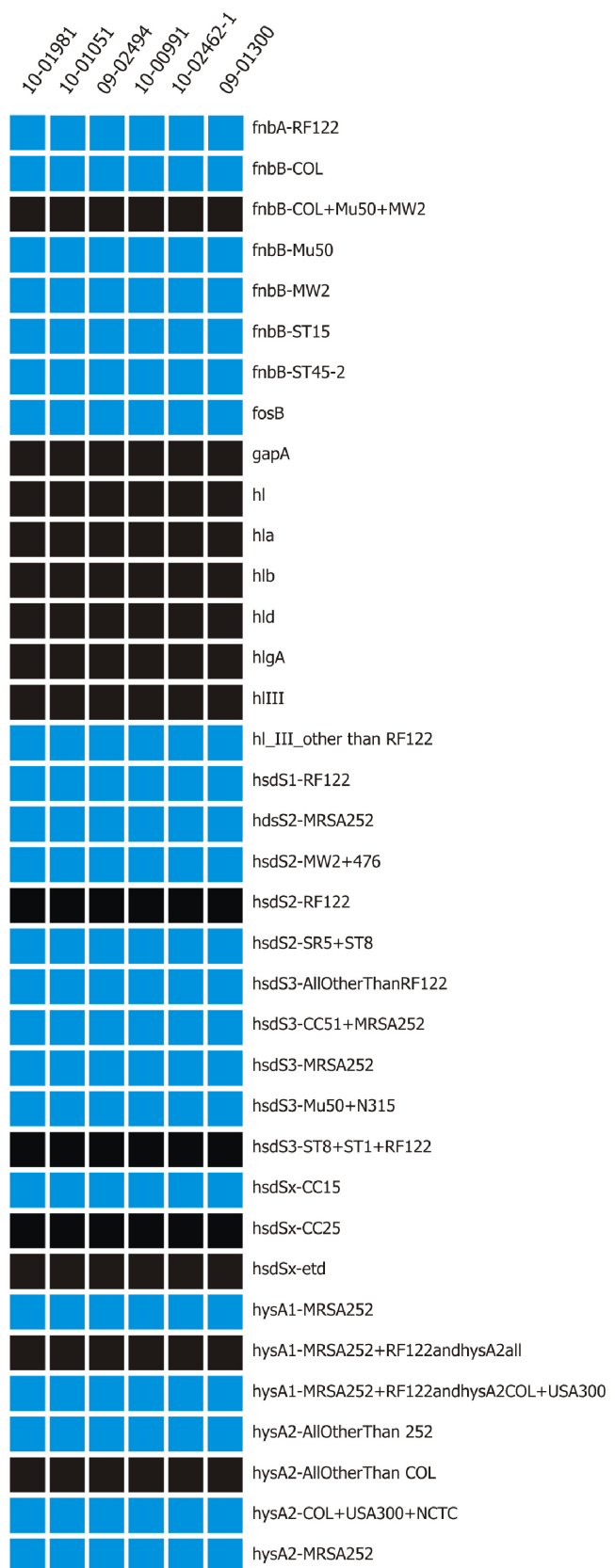

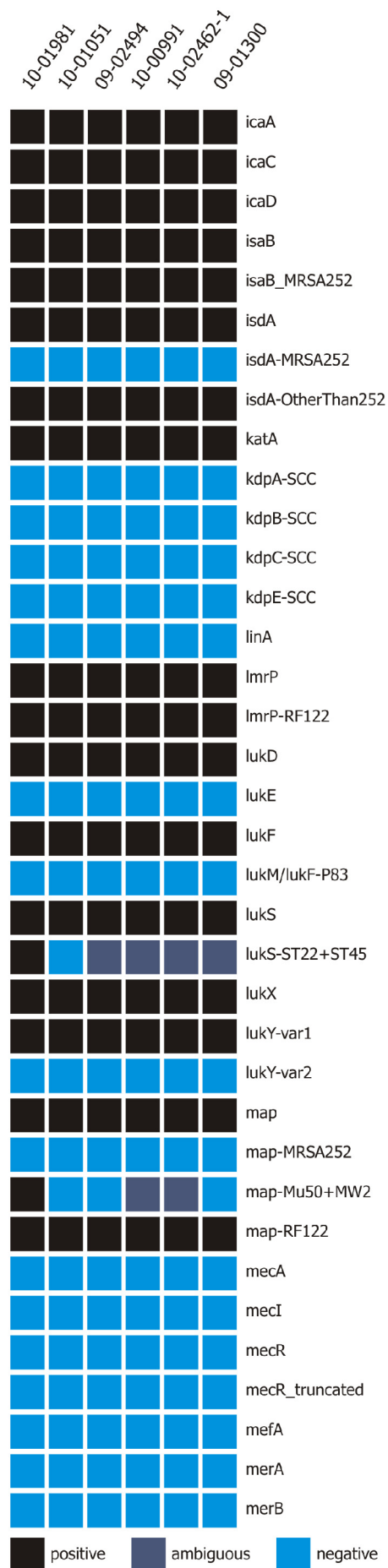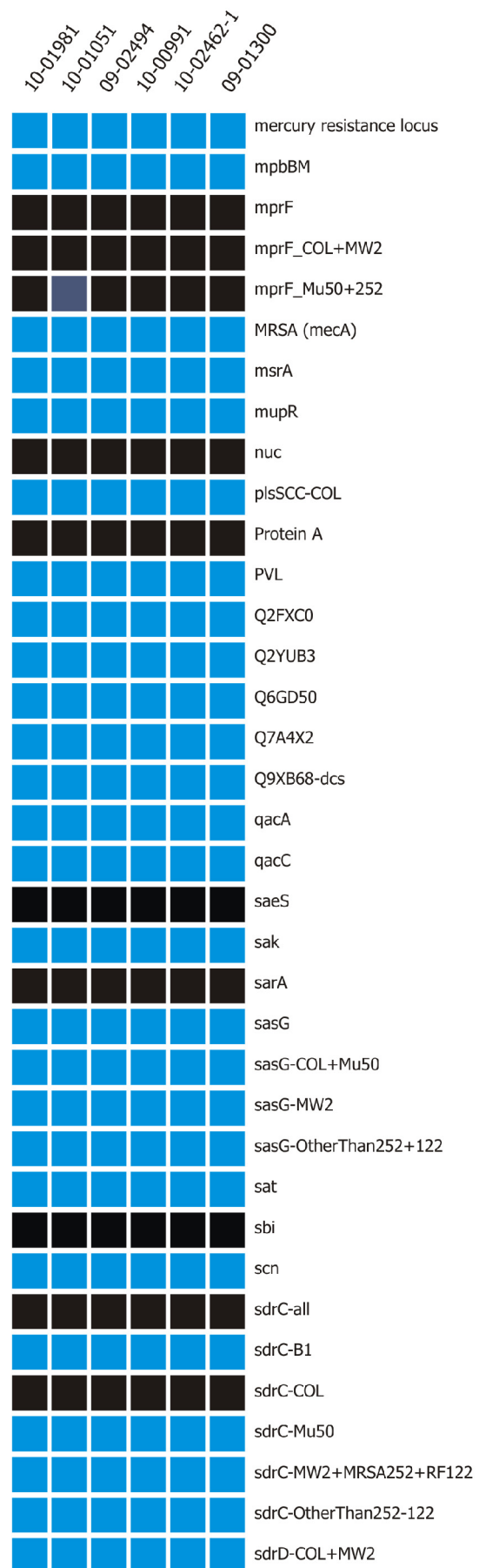

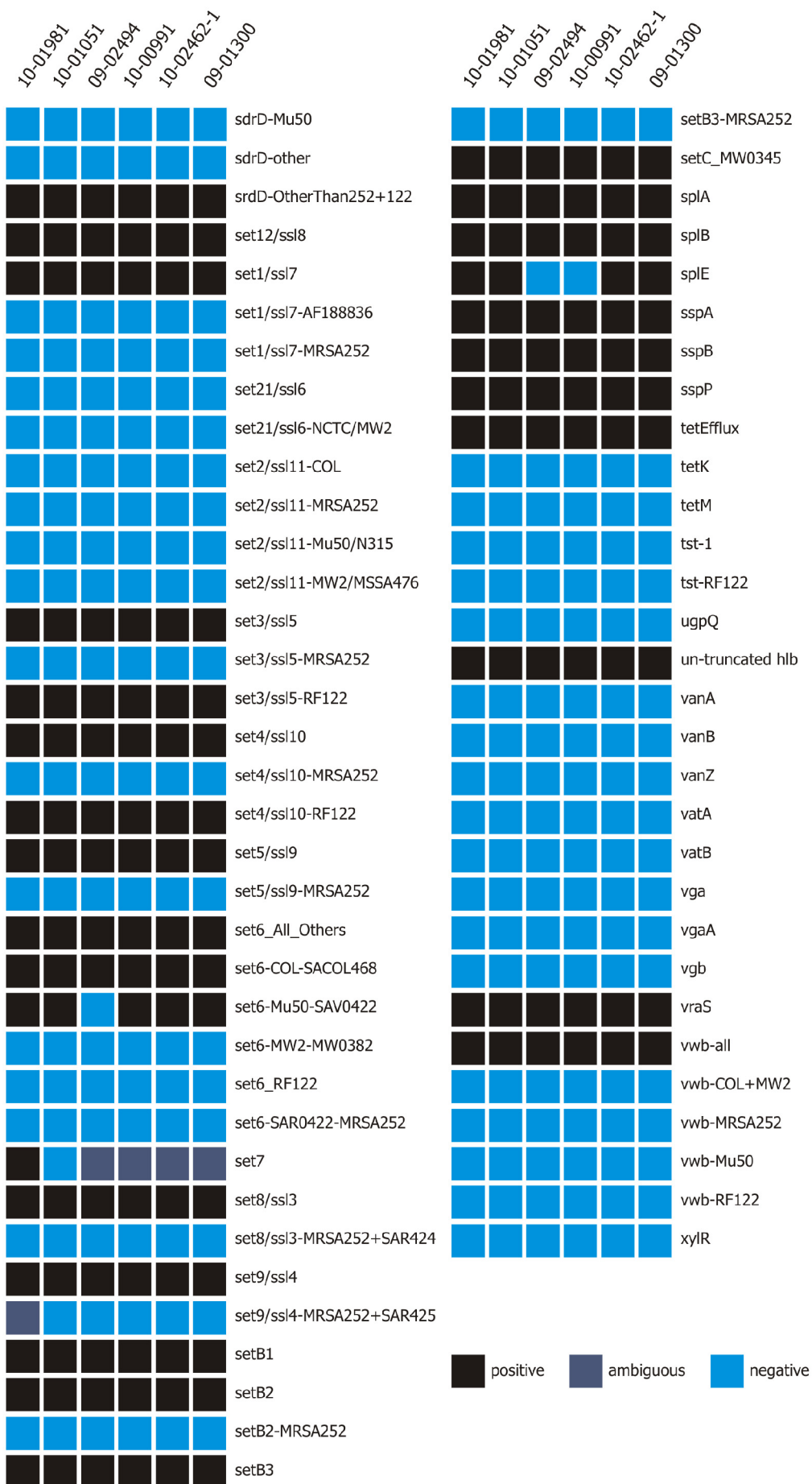

Supplement: Figure S1 — Array hybridisation results for selected isolates. (PDF) [file pone.0024360.s001.pdf]
